# Targeting SIRT1/FoxO3a/Nrf2 and PI3K/AKT Pathways with Rebamipide Attenuates Acetic Acid-Induced Colitis in Rats

**DOI:** 10.3390/ph16040533

**Published:** 2023-04-02

**Authors:** Maha M. Abdel-Fattah, Emad H. M. Hassanein, Ahmed M. Sayed, Shuruq E. Alsufyani, Azza A. K. El-Sheikh, Hany H. Arab, Wafaa R. Mohamed

**Affiliations:** 1 Department of Pharmacology and Toxicology, Faculty of Pharmacy, Beni-Suef University, Beni-Suef 62514, Egypt; 2 Department of Pharmacology & Toxicology, Faculty of Pharmacy, Al-Azhar University, Assiut Branch, Assiut 71524, Egypt; 3 Biochemistry Laboratory, Chemistry Department, Faculty of Science, Assiut University, Assiut 71515, Egypt; 4 Department of Pharmacology and Toxicology, College of Pharmacy, Taif University, P.O. Box 11099, Taif 21944, Saudi Arabia; 5 Basic Health Sciences Department, College of Medicine, Princess Nourah Bint Abdulrahman University, P.O. Box 84428, Riyadh 11671, Saudi Arabia

**Keywords:** rebamipide, ulcerative colitis, SIRT1/FoxO3a, Nrf2/Keap-1, PI3K/AKT

## Abstract

Rebamipide is a quinolone derivative that has been commonly used for the treatment of gastric and duodenal ulcers. However, the molecular mechanisms of rebamipide against acetic acid-evoked colitis have not been adequately examined. Hence, the current study aimed to investigate the ameliorative effect of rebamipide in a rat model of acetic acid-evoked ulcerative colitis and the linked mechanisms pertaining to SIRT1/FoxO3a/Nrf2 and PI3K/AKT pathways. Herein, colitis was induced by the intrarectal administration of 3% acetic acid solution in saline (*v*/*v*) while rebamipide was administered by oral gavage (100 mg/kg/day) for seven days before the colonic insult. The colonic injury was examined by macroscopical and microscopical examination. The current findings demonstrated that rebamipide significantly improved the colonic injury by lowering the colonic disease activity index and macroscopic mucosal injury score. Moreover, it mitigated the histopathological aberrations and microscopical damage score. The favorable outcomes of rebamipide were driven by combating inflammation evidenced by dampening the colonic expression of NF-κBp65 and the pro-inflammatory markers CRP, TNF-α, and IL-6. In the same context, rebamipide curtailed the colonic pro-inflammatory PI3K/AKT pathway as seen by downregulating the immunostaining of PI3K and p-AKT(Ser473) signals. In tandem, rebamipide combated the colonic pro-oxidant events and augmented the antioxidant milieu by significantly diminishing the colonic TBARS and replenishing GSH, SOD, GST, GPx, and CAT. In the same regard, rebamipide stimulated the colonic upstream SIRT1/FoxO3a/Nrf2 axis by upregulating the expression of SIRT1, FoxO3a, and Nrf2, alongside downregulating Keap-1 gene expression. These antioxidant actions were accompanied by upregulation of the protein expression of the cytoprotective signal PPAR-γ in the colons of rats. In conclusion, the present findings suggest that the promising ameliorative features of rebamipide against experimental colitis were driven by combating the colonic inflammatory and oxidative responses. In perspective, augmentation of colonic SIRT1/FoxO3a/Nrf2 and inhibition of PI3K/AKT pathways were engaged in the observed favorable outcomes.

## 1. Introduction

Ulcerative colitis (UC) is a chronic inflammatory illness that occurs on a recurrent basis. UC affects the colorectal mucosa and submucosa, and it is associated with vomiting, recurrent bloody diarrhea, and abdominal pain followed by weight loss. UC causes are unknown; however, various factors such as environmental and genetic factors, microbial pathogens, and up-regulation of the proinflammatory mediators may play a role [1]. It is thought that the colonic inflammatory response and associated cytokines provoke the flaring of irritable bowel disease (IBD) symptoms. Evidence also suggests that diminished antioxidant status and excessive free radical production are linked to UC pathogenesis. In perspective, the reactive oxygen species (ROS) attack the tissue proteins and DNA, resulting in substantial colonic inflammation [2]. 

The inflammatory events play a crucial role in the pathogenesis of UC [3]. In this regard, the nuclear factor kappa B (NF-κB) has a pivotal role by triggering the production of pro-inflammatory cytokines in the colonic microenvironment [4,5,6]. Moreover, evidence has demonstrated the involvement of the phosphoinositide 3-kinase (PI3K)/protein kinase B (AKT) pathway in UC pathogenesis. In fact, PI3K/AKT is engaged in cell differentiation, invasion, and apoptosis signaling [7]. Moreover, PI3K/AKT pathway has been regarded as a classical pro-inflammatory pathway in the pathogenesis of several experimental models of IBD, including UC [3,5,6,8,9,10]. Virtually, stimulation of PI3K/AKT has been associated with NF-κB activation and intensified pro-inflammatory cytokine production, thereby inflicting intestinal mucosal damage [6,8,9]. Generally, suppression of the colonic pro-inflammatory cascades has been regarded as a valid therapeutic strategy for UC management [11,12].

The silent information regulator-1 (SIRT1) protein is a well-studied member of the histone deacetylases that are collectively known as sirtuins. It has been proven to suppress the inflammatory response by inhibiting the NF-κB signal. Recently, it has been reported that SIRT1 plays a role in DNA damage repair, oxidative stress resistance, and cellular lifespan extension. SIRT1 activators have recently been shown to protect against chemically-induced colitis [7,8]. The transcription factor forkhead box O (FoxO) serves as a downstream effector of SIRT1. Generally, FoxOs are crucial for cell survival via transactivating cellular antioxidants including superoxide dismutase (SOD) [13]. In the same regard, the nuclear factor erythroid 2-related factor 2 (Nrf2) regulates the transcription of several genes that control the antioxidative defense system and detoxification process together with regulating the cellular anti-inflammatory mediators [14]. Kelch-like ECH-associated protein-1 (Keap-1) binds Nrf2 tightly, causing its degradation. Upon exposure to cellular stresses, conformational changes in Keap-1 disrupt Keap-1/Nrf2 interaction, preventing Nrf2 degradation [15]. Additionally, the expression of peroxisome proliferator-activated receptor gamma (PPAR-γ) has been reported to be downregulated in UC patients. Evidence exists that PPAR-γ interferes with activators of pro-inflammatory genes such as NF-κB, thereby inhibiting their transcription [16,17]. Several studies have reported that PPAR-γ inhibits the protein expression of diverse pro-inflammatory mediators including the adhesion molecules in the colon of rodents with colitis [18]. Evidence from multiple studies has demonstrated that agents that stimulate SIRT1/FoxO3a/Nrf2 pathway are effective in dampening the flares of colitis [8,13,19]. 

Acetic acid (AA)-induced colitis is an experimental model that shares several features of the human UC including several histopathological findings. In perspective, rodent models of AA-induced colitis revealed diffuse ulceration of the distal colon, alterations in crypt depth, and transmural non-specific inflammatory response [20]. It is a highly reproducible and widely utilized model for searching for novel agents that can attenuate the initiation and progression of UC [21]. The pathogenesis of AA-evoked colitis in rodents involves acid-triggered damage to the colonic mucosa. This event instigates the invasion of colonic mucosa by neutrophils and macrophages and the associated excessive production of pro-inflammatory mediators, culminating in colonic lesion formation [22]. The experimental model of AA-evoked colitis is regarded as a valuable tool in examining the efficacy of several agents for dampening UC manifestations and exploring the associated molecular mechanisms that mediated these favorable effects including inflammation, oxidative stress, and apoptotic events [3,8,21,22,23]. 

**Figure 1:**
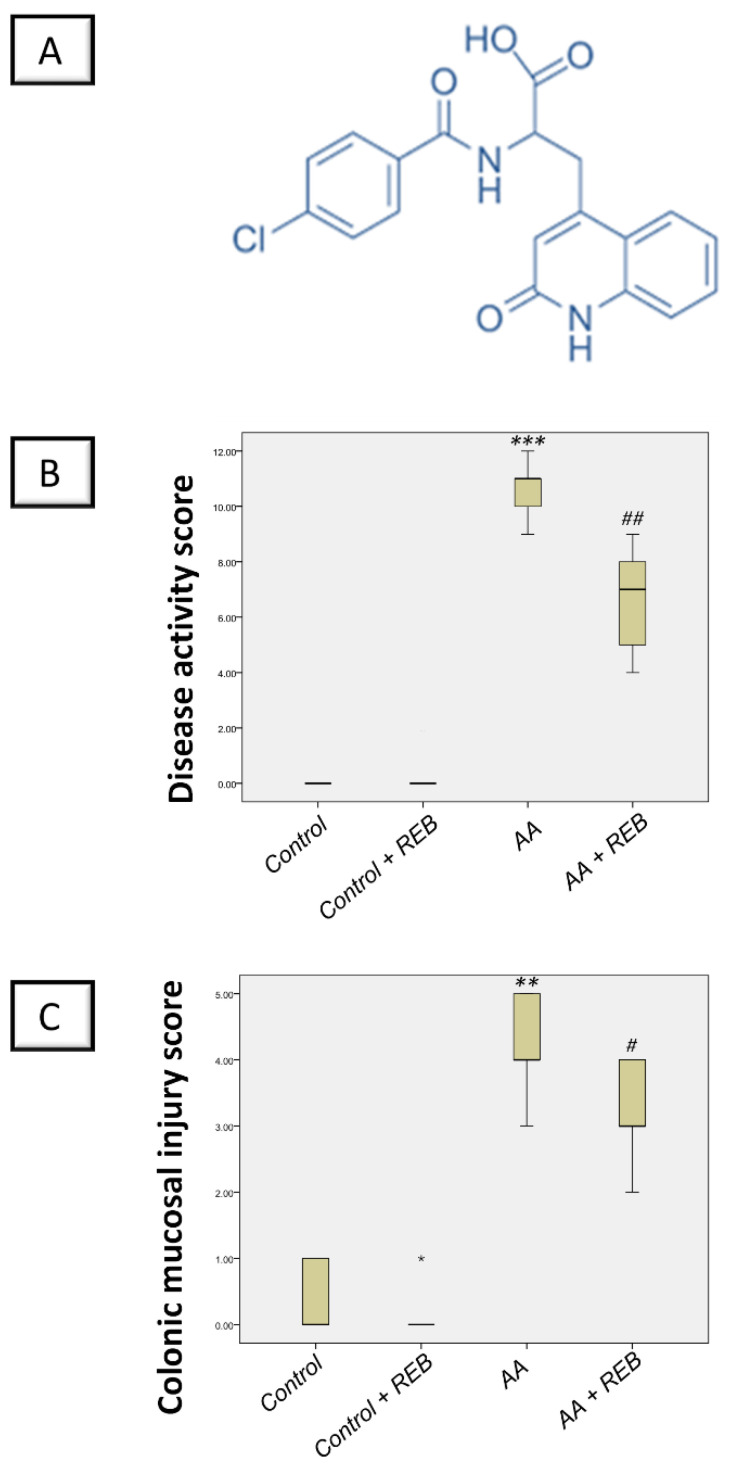
Rebamipide attenuates the colitis severity by lowering scores of the disease activity index and colonic macroscopical mucosal injury in rats. (**A**) The chemical structure of rebamipide. (**B**) The scores of the disease activity index (DAI) summarized by the clinical manifestations of colitis including the degree of weight loss, diarrhea, and rectal bleeding (presence of occult blood). (**C**) The colonic mucosal injury scores (CMIS) characterized by colonic hyperemia, mucosal erosion, and bleeding at the macroscopical level. The scores were expressed as the median with the interquartile range (*n* = 6). The Kruskal–Wallis test was conducted to analyze the significant difference among tested groups followed by the Dunn’s test. ** p < 0.01, or *** p < 0.001, significant from the control group. *^#^ p* < 0.05, or *^##^ p* < 0.01, significant from the colonic injury group. REB, rebamipide; AA, acetic acid.

Rebamipide (REB; the chemical structure is outlined in Figure 1A) is a quinolone derivative that is widely used to treat gastric ulcers [24] by increasing the defensive signals that preserve tissue integrity [25]. The competence of REB to combat ethanol-induced gastric injury has been reported in rodents in vivo [26]. Moreover, REB has been reported to dampen indomethacin-induced damage in gastric epithelial RGM-1 cells in vitro [27]. These favorable effects have been attributed to the marked anti-inflammatory and ROS-scavenging features of REB [28,29]. In addition, stabilization of the mitochondrial bioenergetics and boosting of cellular antioxidants have also advocated the competence of REB to dampen several pathologies including 6-hydroxydopamine-induced Parkinson’s features [30] and ethanol-evoked gastric damage [26]. No previous study has described the efficacy of REB for attenuating AA-induced colitis. Moreover, the molecular underlying mechanisms of REB against colitis have not been adequately examined [31,32]. Hence, the current study was designed to investigate the potential involvement of the antioxidant/cytoprotective SIRT1/FoxO3a/Nrf2 axis and the pro-inflammatory PI3K/AKT pathway in the ameliorative effects of REB against AA-evoked colitis in rats.

## 2. Results

### 2.1. Effect of Rebamipide on the Severity of Colitis and Colonic Macroscopic Pathological Changes in Rats Exposed to Acetic Acid-Induced Colitis

The severity of colonic injury was examined by the disease activity index (DAI) which describes the clinical manifestations associated with colitis. These include the degree of weight loss, consistency of stool (diarrhea), and rectal bleeding (presence of occult blood). In addition, macroscopical examination of the colon was applied and quantified in terms of the colonic mucosal injury score (CMIS). As illustrated in Figure 1B, intrarectal instillation of acetic acid resulted in a significant increase in DAI scores (*p* < 0.001) denoting a marked weight loss, diarrhea, and rectal bleeding versus the control animals. In addition, acetic acid triggered a significant increase in CMIS scores (*p* < 0.01) revealing colonic hyperemia, mucosal erosion, and bleeding versus the control animals (Figure 1C). Rebamipide administration (100 mg/kg; p.o.) to the colonic injury-exposed group counteracted the colonic injury as seen by a significant decrease in DAI scores by 37.5% (*p* < 0.01) and CMIS scores by 28% (*p* < 0.05). These findings indicate the competence of rebamipide for mitigating the colonic injury rendered by acetic acid in animals.

### 2.2. Effect of Rebamipide on the Colonic Histopathological Damage and Microscopical Damage Scores in Rats Exposed to Acetic Acid-Induced Colitis 

**Figure 2:**
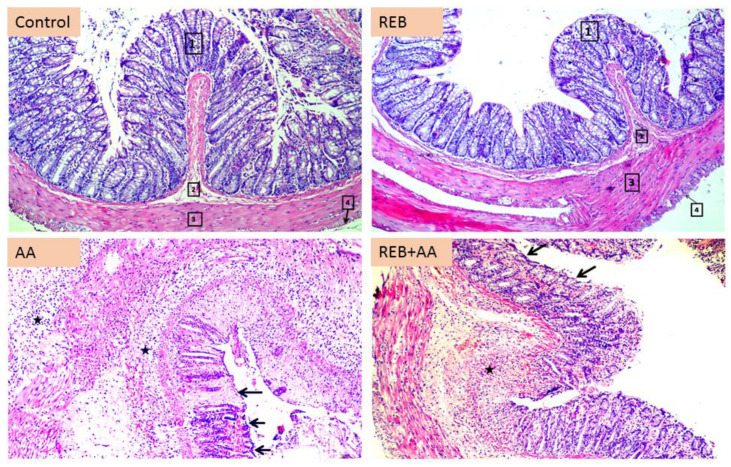
Rebamipide administration counteracts the colonic microscopical damage induced by acetic acid (H–E stain; 100× lower magnification). The photomicrographs at the lower magnification reveal that both the control group and rebamipide (REB)-treated control group demonstrated intact structures of all layers of the colon including the mucosa [1], submucosa [2], muscularis [3], and serosa [4]. On the other hand, the acetic acid (AA) group showed ulcerated surface epithelium (arrow) and severe submucosal infiltration (star). Rebamipide administration to the AA-challenged group attenuated the pathological alterations; however, some colonic areas showed few sites of ulcerated surface epithelium (arrow) and a moderate submucosal infiltration (star). REB, rebamipide; AA, acetic acid.

**Figure 3:**
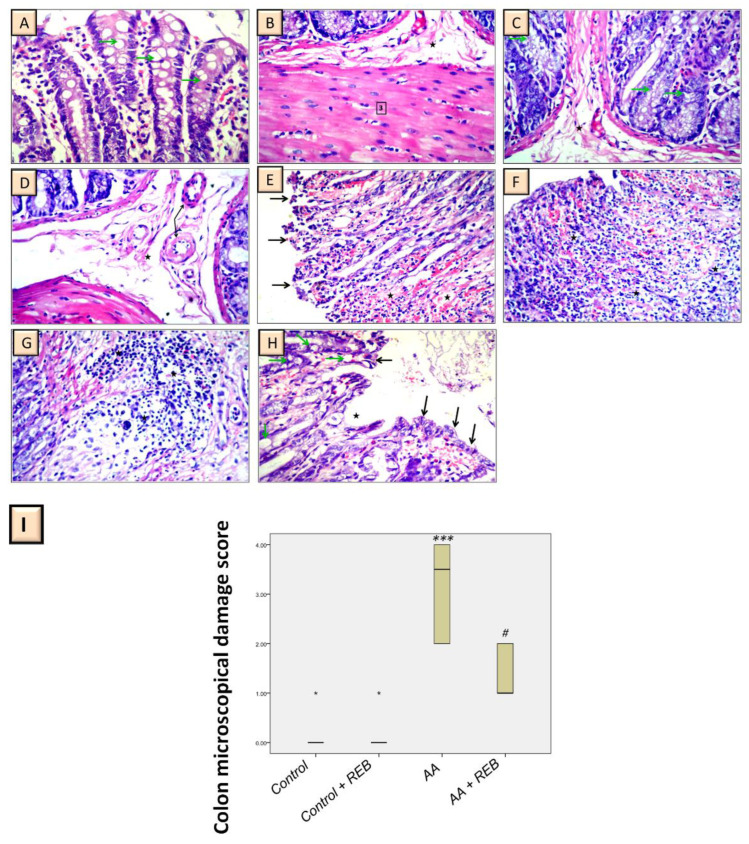
Rebamipide administration attenuates the colonic histopathological aberrations induced by acetic acid (H–E stain; 400× higher magnification). The photomicrographs at higher magnification demonstrated that the control group shows intact goblet cells and intestinal glands (green arrow) (**A**) and a normal picture of the submucosa (star) (**B**). Similar findings for the rebamipide-treated group were also observed as shown by the normal appearance of the submucosa (star) and intestinal gland (green arrow) (**C**) with a normal picture of the submucosa (star) and its blood vessels (arrow) (**D**). On the contrary, the colitis group shows a severe loss of the epithelium (arrow), hemorrhage intermingled with inflammatory cellular infiltration in the mucosa (star) (**E**), severe mixed inflammatory cellular infiltration (star) extending up to the submucosa (**F**), mixed inflammatory cellular infiltration, and micro-abscess formation in the submucosa (star) (**G**). Alternatively, rebamipide administration markedly protected the colon against colitis as indicated by the intact lamina epithelial cells (arrow), and few ulcers (star) (**H**). (**I**) The injury in the colonic sections was quantified as the colon microscopical damage scores. The scores were expressed as the median with the interquartile range (*n* = 6). The Kruskal–Wallis test was conducted to analyze the significant difference among tested groups followed by the Dunn’s test. *** p < 0.001, significant from the control group. *^#^ p* < 0.05, significant from the colonic injury group. REB, rebamipide; AA, acetic acid.

To further reinforce the ameliorative potential of rebamipide on colitis development, examination of the colon cross-sections was performed using hematoxylin and eosin (H–E) staining. As illustrated in Figure 2 which depicts the lower magnification pics (upper panels) and Figure 3A–D (higher magnification), the control rats (Figure 3A,B) and control-treated-rebamipide animals (Figure 3C,D) showed a normal colon architecture seen by normal layers of the colon, intact intestinal glands, normal goblet cell appearance, and normal mucosal layer appearance with intact normal blood vessels. On the other hand, the colon specimens obtained from acetic acid-challenged rats revealed several histological changes characteristic of UC including an ulcerated surface epithelium, submucosal leukocyte infiltration, total loss of the epithelium, hemorrhage intermingled with inflammatory cellular infiltration in the mucosa extending up to the submucosa, goblet cell exhaustion, and micro-abscess formation in the submucosa (Figure 2 and Figure 3E–G). Interestingly, rebamipide administration (100 mg/kg; p.o.) to the colonic injury-exposed group attenuated the histopathological aberrations rendered by acetic acid as illustrated by intact epithelial cells of the lamina, and intact goblet cell architecture in intestinal glands. In some colon areas, mild ulcerated surface epithelium, mild submucosal leukocyte infiltration, and few ulcers were observed (Figure 2 and Figure 3H).

The injury in the colonic sections was quantified as the colon microscopical damage scores which characterize the surface epithelial ulceration, hemorrhage, inflammatory cell influx, and goblet cell depletion in intestinal glands. As illustrated in Figure 3I, a significant increase in the microscopical damage scores (*p* < 0.001) was observed in the colonic injury group versus the control animals. Rebamipide administration (100 mg/kg; p.o.) to the colonic injury-exposed group counteracted the colonic injury as seen by a significant decrease in the damage scores by 57.9% (*p* < 0.05). These findings reinforce the competence of rebamipide for lowering the colonic injury rendered by acetic acid at the microscopical level in animals.

### 2.3. Effect of Rebamipide on the Inflammatory Signals and Colonic PI3K/AKT Pathway in Rats Exposed to Acetic Acid-Induced Colitis 

**Figure 4:**
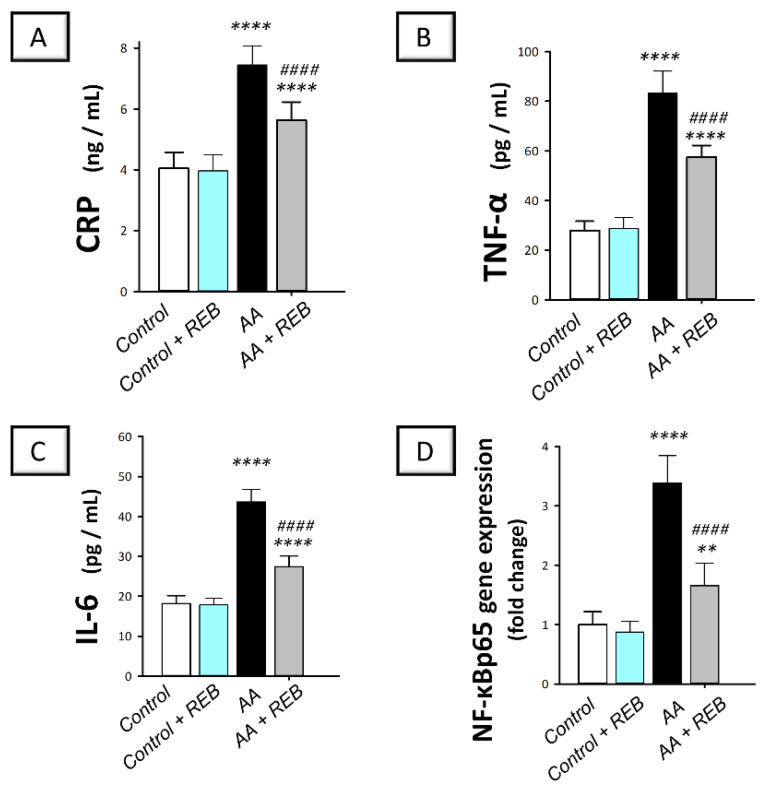
Rebamipide inhibits the pro-inflammatory signals in rats exposed to acetic acid-induced colitis. (**A**) Serum C-reactive protein (CRP) levels. (**B**) Serum tumor necrosis factor-alpha (TNF-α) levels. (**C**) Serum interleukin-6 (IL-6) levels. (**D**) The gene expression levels of the nuclear factor kappa B (NF-κBp65) in the colonic tissue. Each bar represents the mean ± S.D. (*n* = 7–8). A one-way ANOVA statistical test was conducted to analyze the significant difference among tested groups followed by a Tukey’s test. ** p < 0.01, or **** p < 0.0001, significant from the control group. ^#*###*^ *p* < 0.0001, significant from the colonic injury group. REB, rebamipide; AA, acetic acid.

The pro-inflammatory response of rats challenged with acetic acid was explored by measuring serum C-reactive protein (CRP), tumor necrosis factor-alpha (TNF-α), and interleukin-6 (IL-6) in addition to the colonic gene expression of the nuclear factor kappa B (NF-κBp65). In the same regard, the inflammation-associated phosphoinositide-3 kinase (PI3K)/protein kinase B (AKT) pathway was examined by detecting the immunostaining of PI3K and the phosphorylated form of AKT (p-AKT(Ser473)) in the colonic tissue. As illustrated in Figure 4A–C, intrarectal instillation of acetic acid resulted in a significant increase (*p* < 0.0001) in serum CRP, TNF-α, and IL-6 by 83.9%, 198%, and 140.6%, respectively, versus the control animals. In addition, acetic acid triggered a significant increase in the colonic gene expression of NF-κBp65 (*p* < 0.0001) by 239.1% versus the control animals (Figure 4D). Rebamipide administration (100 mg/kg; p.o.) to the colonic injury-exposed group dampened the pro-inflammatory response as seen by a significant decrease (*p* < 0.0001) in serum CRP, TNF-α, and IL-6 by 24.4%, 30.9%, and 37.2%, respectively, reflecting the mitigation of systemic inflammation. Moreover, it significantly downregulated the gene expression of the pro-inflammatory NF-κBp65 (*p* < 0.0001) by 51.1%, versus the colonic injury group, confirming its inhibition locally in the colonic tissues. 

**Figure 5:**
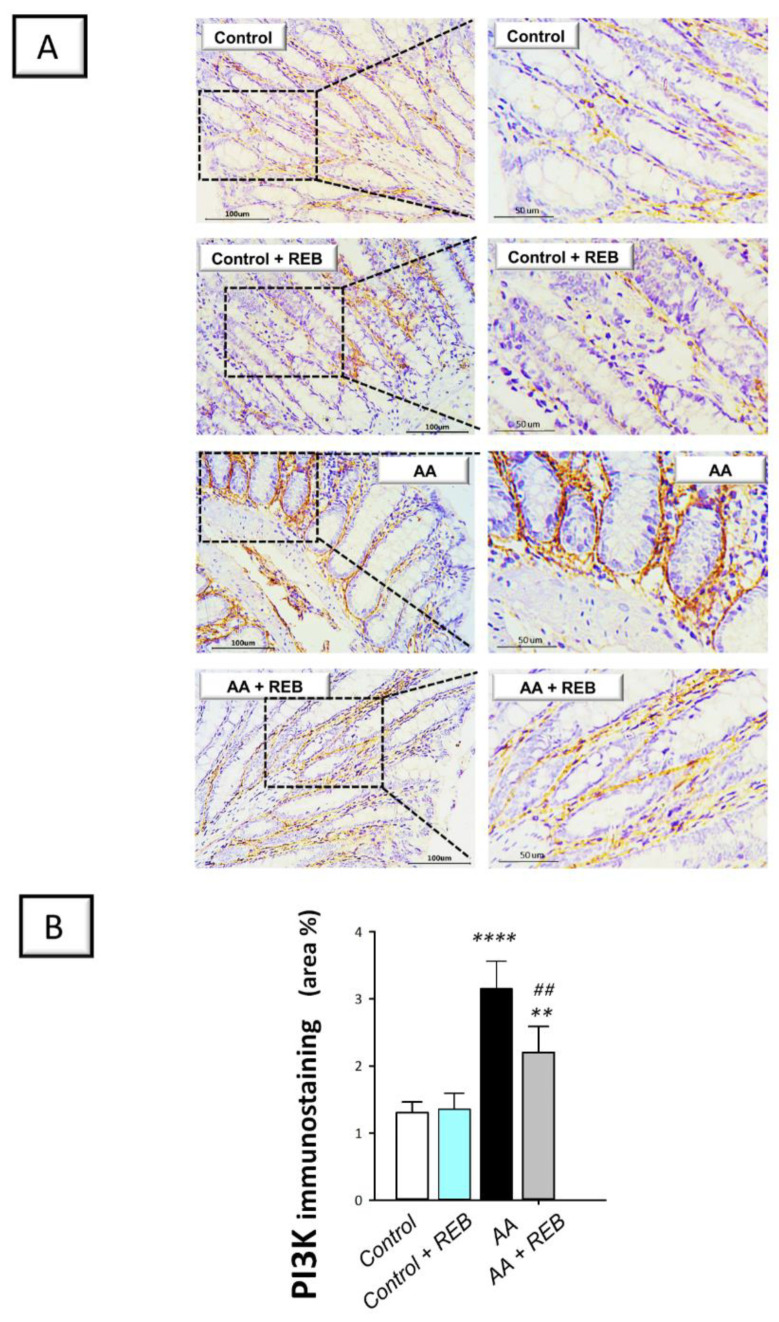
Rebamipide inhibits the colonic PI3K/AKT pathway in rats exposed to acetic acid-induced colitis. (**A**) Immunostaining of the phosphoinositide-3 kinase (PI3K) pro-inflammatory signal in the colonic tissue. (**B**) Quantification of the colonic PI3K immunostaining. Each bar represents the mean ± S.D. (*n* = 6). A one-way ANOVA statistical test was conducted to analyze the significant difference among tested groups followed by a Tukey’s test. ** p < 0.01, or **** p < 0.0001, significant from the control group. *^##^ p* < 0.01, significant from the colonic injury group. REB, rebamipide; AA, acetic acid.

**Figure 6:**
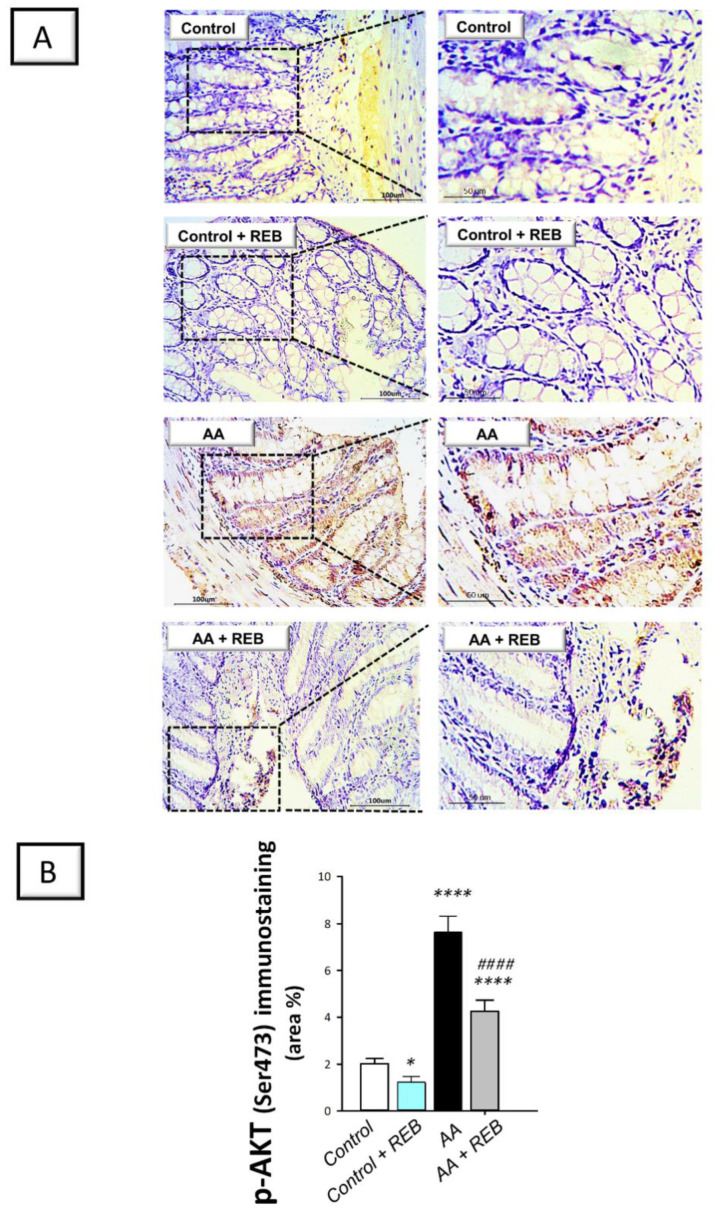
Rebamipide inhibits the colonic PI3K/AKT pathway in rats exposed to acetic acid-induced colitis. (**A**) Immunostaining of the phosphorylated form of protein kinase B (p-AKT(Ser473)) in the colonic tissue. (**B**) Quantification of colonic p-AKT(Ser473) immunostaining. Each bar represents the mean ± S.D. (*n* = 6). A one-way ANOVA statistical test was conducted to analyze the significant difference among tested groups followed by a Tukey’s test. * p < 0.05, or **** p < 0.0001, significant from the control group. ^#*###*^ *p* < 0.0001, significant from the colonic injury group. REB, rebamipide; AA, acetic acid.

In the context of colonic pro-inflammatory events, the PI3K/AKT pathway was also investigated as a pertinent pro-inflammatory signal in experimental models of colitis [5,6]. As illustrated in Figure 5B and Figure 6B, intrarectal instillation of acetic acid resulted in a significant increase (*p* < 0.0001) in the colonic immunostaining of PI3K and p-AKT by 142.1%, and 279.5%, respectively, versus the control animals. Rebamipide administration (100 mg/kg; p.o.) to the colonic injury-exposed group inhibited the PI3K/AKT pathway as seen by a significant decrease in the colonic immunostaining of PI3K (*p* < 0.01) and p-AKT (*p* < 0.0001) by 30.2%, and 44.2%, respectively. Together these findings reveal the competence of rebamipide for dampening the pro-inflammatory signals and curbing the colonic PI3K/AKT pathway in rats exposed to acetic acid-induced colitis. 

### 2.4. Effect of Rebamipide on the Colonic Oxidative Stress Markers and Antioxidant Defenses in Rats Exposed to Acetic Acid-Induced Colitis 

**Figure 7:**
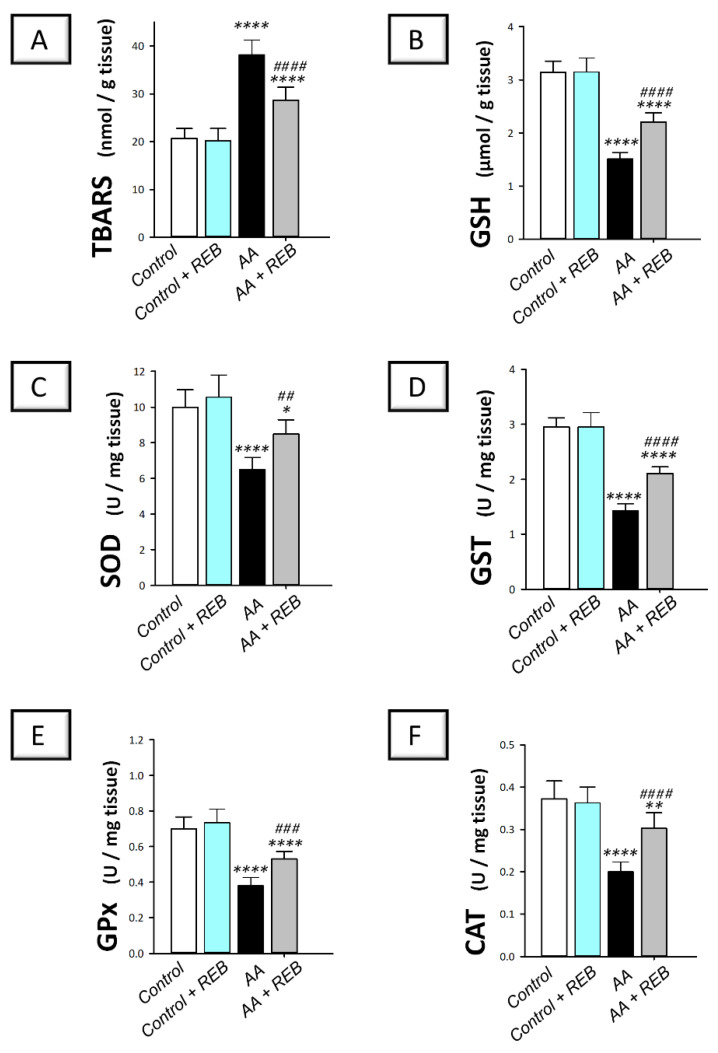
Rebamipide inhibits the colonic pro-oxidant signals and augments the antioxidant defenses in rats exposed to acetic acid-induced colitis. (**A**) Colonic lipid peroxides were measured as thiobarbituric acid reactive substances (TBARS). (**B**) Colonic reduced glutathione (GSH) levels. (**C**) Colonic superoxide dismutase (SOD) activity. (**D**) Colonic glutathione-S-transferase (GST) activity. (**E**) Colonic glutathione peroxidase (GPx) activity. (**F**) Colonic catalase (CAT) activity. Each bar represents the mean ± S.D. (*n* = 8). A one-way ANOVA statistical test was conducted to analyze the significant difference among tested groups followed by a Tukey’s test. ** p < 0.01, or * p < 0.05, or **** p < 0.0001, significant from the control group. *^##^ p* < 0.01, *^###^ p* < 0.001, or ^#*###*^ *p* < 0.0001, significant from the colonic injury group. REB, rebamipide; AA, acetic acid.

The colonic pro-oxidant milieu of rats challenged with acetic acid-induced colitis was explored by measuring the pro-oxidant lipid peroxides (measured as thiobarbituric acid reactive substances; TBARS) and the colonic antioxidant defenses including the reduced form of glutathione (GSH) alongside the activities of superoxide dismutase (SOD), glutathione-S-transferase (GST), glutathione peroxidase (GPx), and catalase (CAT). As illustrated in Figure 7A–C, intrarectal instillation of acetic acid resulted in a significant increase in the colonic TBARS (*p* < 0.0001) by 85.1% together with a significant decline (*p* < 0.0001) in the colonic GSH and SOD by 51.9% and 35%, respectively, versus the control animals. Moreover, acetic acid triggered a significant decrease (*p* < 0.0001) in the activities of GST, GPx, and CAT by 51.7%, 45.8%, and 46.3%, respectively, versus the control animals (Figure 7D–F). Rebamipide administration (100 mg/kg; p.o.) to the colonic injury-exposed group dampened the colonic pro-oxidant response seen by a significant decrease in TBARS (*p* < 0.0001) by 24.9% together with a significant augmentation of the colonic antioxidant defenses including GSH, (*p* < 0.0001), SOD (*p* < 0.01), GST (*p* < 0.0001), GPx (*p* < 0.001), and CAT (*p* < 0.0001) by 45.7%, 30.5%, 47.8%, 39.5%, and 51.3%, respectively, versus the colonic injury group. These findings reveal the competence of rebamipide for dampening the pro-oxidant signals and augmenting the colonic antioxidant defenses in rats exposed to acetic acid-induced colitis.

### 2.5. Effect of Rebamipide on the Colonic Antioxidant Nrf2/Keap-1 Pathway and the Cytoprotective Signal PPAR-γ in Rats Exposed to Acetic Acid-Induced Colitis

**Figure 8:**
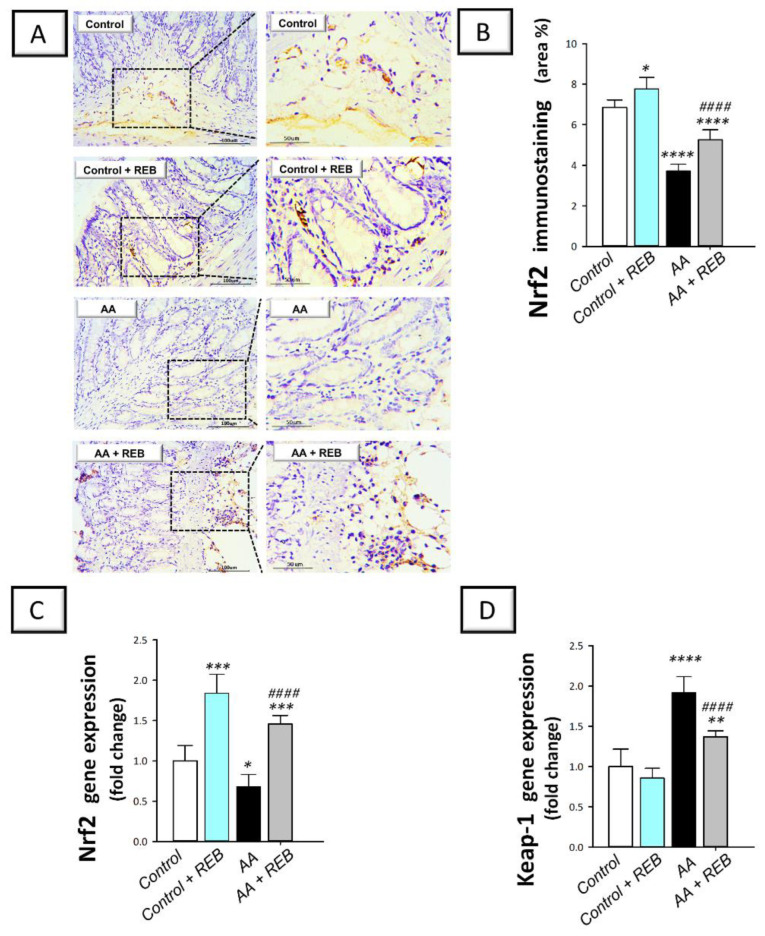
Rebamipide stimulates the colonic Nrf2/Keap-1 pathway and augments the cytoprotective signal PPAR-γ in rats exposed to acetic acid-induced colitis. (**A**) Immunostaining of the nuclear factor erythroid 2-related factor 2 (Nrf2) in the colonic tissue. (**B**) Quantification of colonic Nrf2 immunostaining. (**C**) Gene expression levels of Nrf2 in the colonic tissue. (**D**) Gene expression levels of Kelch-like ECH-associated protein-1 (Keap-1) in the colonic tissue. Each bar represents the mean ± S.D. (*n* = 6 for Nrf2 immunostaining and *n* = 7 for the gene expression data). A one-way ANOVA statistical test was conducted to analyze the significant difference among tested groups followed by a Tukey’s test. * p < 0.05, ** p < 0.01, *** p < 0.001, or **** p < 0.0001, significant from the control group. ^#*###*^ *p* < 0.0001, significant from the colonic injury group. REB, rebamipide; AA, acetic acid.

**Figure 9:**
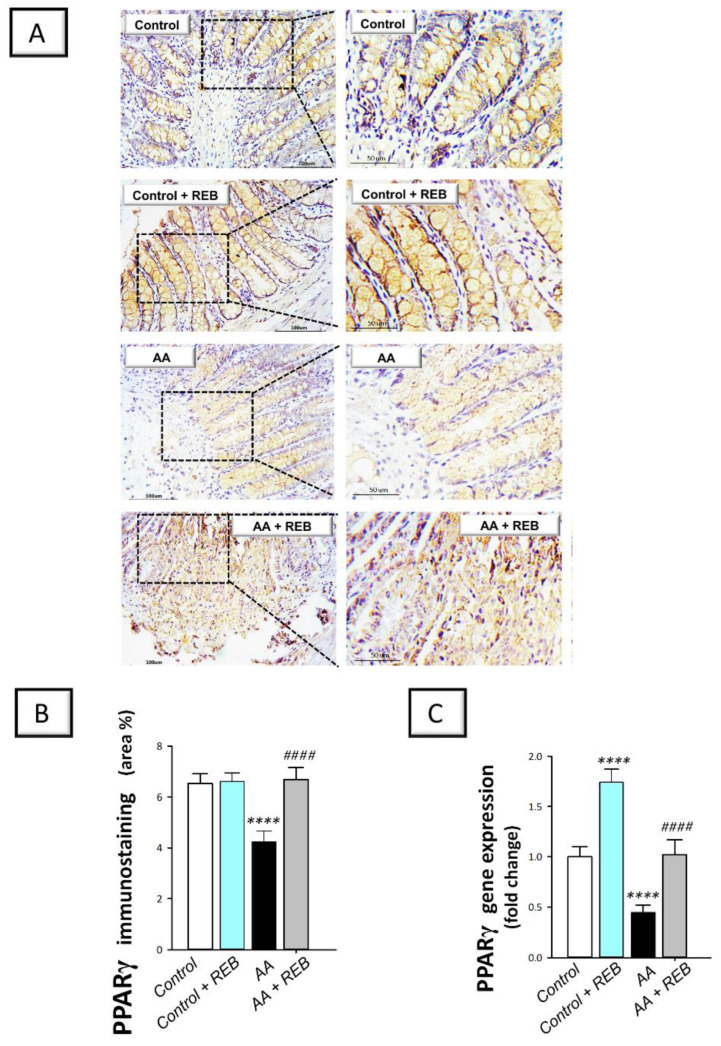
Rebamipide replenishes the colonic cytoprotective signal PPAR-γ in rats exposed to acetic acid-induced colitis. (**A**) Immunostaining of the peroxisome proliferator-activated receptor gamma (PPAR-γ) in the colonic tissue. (**B**) Quantification of colonic PPAR-γ immunostaining. (**C**) Gene expression levels of PPAR-γ in the colonic tissue. Each bar represents the mean ± S.D. (*n* = 6). A one-way ANOVA statistical test was conducted to analyze the significant difference among tested groups followed by a Tukey’s test. **** p < 0.0001, significant from the control group. ^#*###*^ *p* < 0.0001, significant from the colonic injury group. REB, rebamipide; AA, acetic acid.

The colonic antioxidant milieu of rats challenged with acetic acid-induced colitis was further characterized by exploring the colonic *Nrf2/Keap-1 pathway* by detecting the immunostaining of the nuclear factor erythroid 2-related factor 2 (Nrf2) and the gene expression of its inhibitory unit Kelch-like ECH-associated protein-1 (Keap-1). Moreover, the immunostaining of the cytoprotective signal peroxisome proliferator-activated receptor gamma (PPAR-γ) was detected in the colonic tissue. As illustrated in Figure 8A–C, intrarectal instillation of acetic acid resulted in a significant decline in the colonic Nrf2 immunostaining (*p* < 0.0001) and gene expression (*p* < 0.05) by 45.8% and 32%, respectively, together with a significant increase in the gene expression of the colonic Keap-1 (*p* < 0.0001) by 91.9%, versus the control animals. Moreover, acetic acid triggered a significant decrease (*p* < 0.0001) in the colonic PPAR-γ immunostaining and gene expression by 35.3% and 55%, respectively, versus the control animals (Figure 9B,C). Rebamipide administration (100 mg/kg; p.o.) to the colonic injury-exposed group stimulated Nrf2/Keap-1 pathway as seen by a significant increase (*p* < 0.0001) in Nrf2 immunostaining and gene expression by 41.4% and 114.3%, respectively, alongside a significant decrease in Keap-1 gene expression (*p* < 0.0001) by 28.6%. In the same regard, rebamipide administration replenished PPAR-γ levels as seen by a significant increase (*p* < 0.0001) in its immunostaining and gene expression by 58.1% and 126.8%, respectively, versus the colonic injury group. These findings reveal the competence of rebamipide for activating the colonic Nrf2/Keap-1 pathway and replenishing the cytoprotective PPAR-γ in rats exposed to acetic acid-induced colitis.

### 2.6. Effect of Rebamipide on the Colonic Antioxidant SIRT1/FoxO3a Pathway in Rats Exposed to Acetic Acid-Induced Colitis 

**Figure 10:**
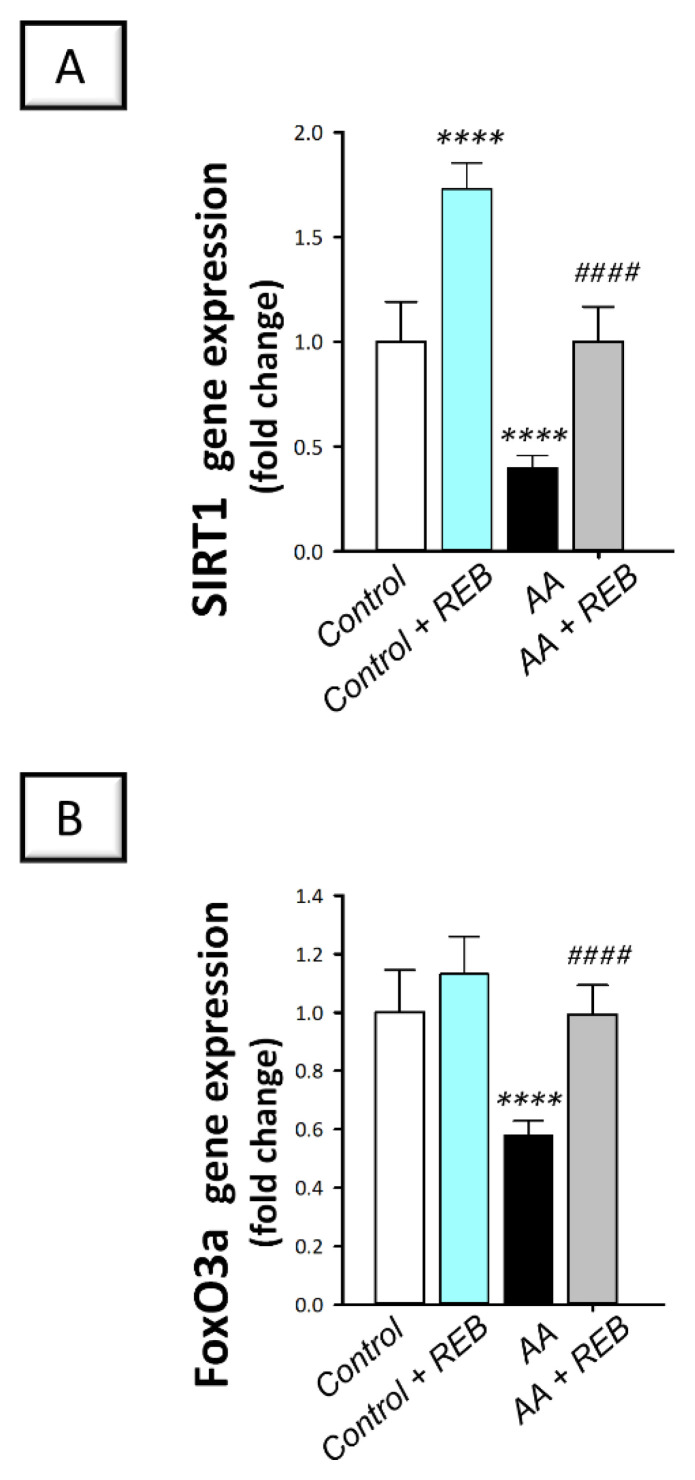
Rebamipide activates the colonic antioxidant SIRT1/FoxO3a pathway in rats exposed to acetic acid-induced colitis. (**A**) The gene expression levels of the silent information regulator 1 (SIRT1) in the colonic tissue. (**B**) The gene expression levels of forkhead box O3a (FoxO3a) in the colonic tissue. Each bar represents the mean ± S.D. (*n* = 6). A one-way ANOVA statistical test was conducted to analyze the significant difference among tested groups followed by a Tukey’s test. **** p < 0.0001, significant from the control group. ^#*###*^ *p* < 0.0001, significant from the colonic injury group. REB, rebamipide; AA, acetic acid.

The colonic antioxidant SIRT1/FoxO3a pathway was explored by detecting the colonic gene expression of the silent information regulator-1 (SIRT1) and forkhead box 3a (FoxO3a) antioxidant/cytoprotective signals. As illustrated in Figure 10A,B, intrarectal instillation of acetic acid resulted in a significant decline (*p* < 0.0001) in the colonic gene expression of SIRT1 and FoxO3a by 60% and 42.2%, respectively, versus the control animals. Rebamipide administration (100 mg/kg; p.o.) to the colonic injury-exposed group stimulated the SIRT1/FoxO3a pathway as seen by a significant increase (*p* < 0.0001) in the gene expression of SIRT1 and FoxO3a by 150.1% and 71.5%, respectively, versus the colonic injury group. These findings reveal the competence of rebamipide for activating the colonic SIRT1/FoxO3a pathway in rats exposed to acetic acid-induced colitis. 

## 3. Discussion

**Figure 11:**
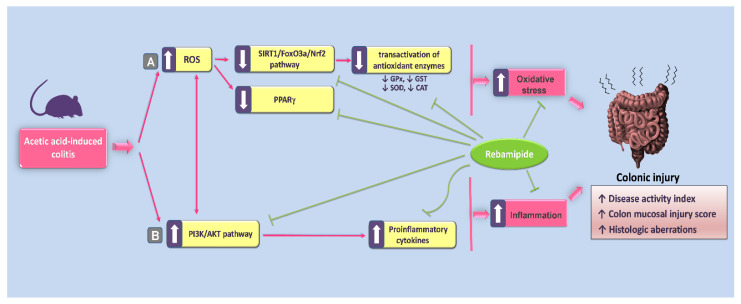
Summary of the proposed underlying molecular mechanisms by which rebamipide attenuates acetic acid-evoked colonic injury. The mechanisms for the beneficial effects of rebamipide against colitis can be summarized as follows: (**A**) Rebamipide curtails the oxidative stress in the colonic microenvironment by counteracting acetic acid-induced inhibition of the silent information regulator-1 (SIRT1)/forkhead box O3a (FoxO3a)/nuclear factor erythroid 2-related factor 2 (Nrf2) pathway and acetic acid-induced depletion of the peroxisome proliferator-activated receptor gamma (PPAR-γ) cytoprotective signal. (**B**) Meanwhile, rebamipide suppressed the inflammatory signals/cytokines by suppressing acetic acid-induced activation of the phosphoinositide 3-kinase (PI3K)/protein kinase B (AKT) pro-inflammatory pathway. In the context of IBD pathogenesis, suppression of the colonic SIRT1/FoxO3a/Nrf2 axis has been previously characterized in experimental IBD models where excessive reactive oxygen species (ROS) generation is associated with lowered expression of SIRT1, FoxO3a, Nrf2, and HO-1 and exaggerated oxidative stress status in the colonic microenvironment [7,23,34,35]. The solid arrow represents activation; the blunt arrow represents inhibition.

Ulcerative colitis (UC) is a chronic inflammatory disorder manifested by aggressive colon and rectal mucosal inflammation. Currently, the approaches being used for the clinical management of UC have various side effects and even lowered responsiveness in many cases [33]. Consequently, there is a constant desire to find new therapies that can be used to control UC. Rebamipide (REB) has demonstrated a protective impact against dextran sulfate sodium-induced colitis. However, no previous work investigated the impact of rebamipide against acetic acid (AA)-induced UC and the underlying mechanisms. Herein, the current study reports the beneficial effects of rebamipide against AA-induced UC that were driven by suppressing the colonic inflammatory response and oxidative stress mainly via curbing PI3K/AKT pathway alongside stimulating the antioxidant SIRT1/FoxO3a/Nrf2 axis and PPAR-γ cytoprotective signal, resulting in improved UC manifestations (Figure 11).

Herein, AA was used in the current study for UC induction in rats. Ample evidence exists that the AA-induced colitis model mimics the pathological picture in human UC [21]. In the current set of experiments, the AA-induced colon injury was confirmed by the increased DAI and CMIS compared to control rats. Interestingly, these effects were counteracted by REB administration. These findings were further supported by the ability of REB to attenuate AA-induced histological aberrations as proven by attenuated disruption of the colonic mucosa, dampened leukocyte influx into the submucosa, and intact goblet cell architecture in intestinal glands. These findings concur with earlier research that revealed the protective effects of REB against experimental gastrointestinal damage [31,32]. 

Inflammation has been proven to be a key player in colitis development. Our data revealed a significant upregulation of serum pro-inflammatory biomarkers in AA-induced UC, as revealed by the significant elevation of serum CRP, TNF-α, and IL-6 with upregulated colonic NF-κBp65 expression, compared to the control rats. These findings are consistent with the previous literature [36]. In fact, activation of NF-κB has been reported to upregulate the expression of several pro-inflammatory cytokines including TNF-α and IL-6 in macrophages and epithelial cells [10,37]. In the context of intestinal inflammation, TNF-α has a crucial role in colitis pathogenesis as it induces ROS production and provokes epithelial cell apoptosis. In addition, TNF-α instigates diverse inflammatory changes through the activation of the NF-κB pathway [38]. In UC patients, the clinical severity of UC has been correlated with serum TNF-α levels. Herein, REB was found to exert considerable anti-inflammatory effects against AA-induced UC by curtailing CRP, TNF-α, and IL-6 together with downregulating NF-κBp65 expression compared to the AA group. Consistently, the marked anti-inflammatory features of REB have been previously characterized in several experimental pathologies [39,40]. 

The current study evaluated the possible involvement of the classical inflammatory PI3K/AKT pathway in REB’s protection against AA-induced colitis. Virtually, the PI3K/AKT pathway is a key player in several cellular activities and has been proven to engage in UC progression and development [7,9]. In perspective, PI3K/AKT pathway has been linked to the production of several proinflammatory cytokines, thereby triggering colonic mucosal damage [5]. The current data showed that AA significantly upregulated PI3K and p-AKT(Ser473), thereby intensifying the pro-inflammatory events in the colonic microenvironment. These data are in line with the previous literature that reported the increased protein expression of PI3K and p-AKT in experimental colitis models in vivo, including AA-induced colitis [3,5,6,8,9,10] and LPS-treated intestinal myofribroblast CCD-18Co cells in vitro [10]. In the context of inflammatory events, stimulation of PI3K/AKT pathway has been associated with the activation of NF-κB, culminating in an exaggerated inflammatory response. This can be attributed to the fact that AKT activates the inhibitor of NF-κB (IκB) kinase (IKK) resulting in the degradation of the inhibitory unit IκB. Hence, the liberation of activated NF-κBp65 occurs together with the associated production of pro-inflammatory cytokines such as TNF-α and IL-6 [9,21]. Of note, the present findings of activated PI3K/AKT pathway in rats with AA-evoked colitis are contrary to the data reported in an experimental model of dextran sodium sulfate-induced colitis in mice [7]. The discrepancy could be attributed to the difference in the animal species, nature of the experimental model, and severity of the colonic injury. Herein, the administration of REB significantly downregulated PI3K and p-AKT protein expression; pointing to its beneficial role in mitigating AA-evoked colonic injury. Ample evidence demonstrated that inhibition of PI3K/AKT pathway and the linked pro-inflammatory responses is regarded as an effective tool for dampening the severity of UC and protecting intestinal cells [3,5,6,9,10]. In the same context, the inhibition of the mammalian target of rapamycin (mTOR), a downstream molecule of PI3K/AKT pathway, has also been proven to afford significant anti-inflammatory actions against colitis progression through dampening the T-cell function and NF-κB activation. Thus, the interventions that trigger PI3K/AKT abrogation in colitis have been proven as effective modalities in colitis management [21,41]. 

Studies have demonstrated the involvement of oxidative stress in the pathogenesis of IBD experimentally and clinically [42]. In this regard, activated phagocytes trigger robust ROS production, resulting in excessive lipid peroxidation and colonic injury [2,42]. The oxidative stress and depletion of the antioxidant machinery provoke an imbalance in the colonic redox milieu. The current data revealed that AA-induced colitis was accompanied by a notable increase in TBARS content and significantly reduced colon antioxidants as proven by the decreased GSH content and SOD, GST, GPx, and CAT activities. These findings concur with the previous literature that reported exaggerated oxidative stress and depletion of the colonic antioxidant defenses in experimental models of colitis [7]. Herein, REB caused a significant reduction in colon TBARS content and augmentation of colonic GSH, SOD, GST, GPx, and CAT. In fact, REB‘s antioxidant features have been documented in several experimental pathologies [30], including indomethacin-evoked gastric injury and impaired spermatogenesis models [27,43,44]. Moreover, the competence of REB in boosting the cellular antioxidant defenses [26] and scavenging free radicals has been previously reported [45]. 

SIRT1, a redox-sensitive NAD-dependent histone deacetylase, is a cytoprotective cellular signal involved in the regulation of cell physiology and survival. Ample evidence has revealed that SIRT1 depletion is engaged in the pathogenies of inflammatory diseases including ulcerative colitis. Notably, inhibition of colonic SIRT1/FoxO3a/Nrf2 has been reported in experimental models of IBD resulting in exaggerated oxidative stress and marked mucosal injury [7,23,34,35]. Evidence has also indicated that downregulated expression of SIRT1 triggers exaggerated pro-inflammatory events and colonic damage [8]. In this context, the knockout of intestinal *SIRT1* has been linked to aberrant activation of intestinal inflammation, TNF-α spike, and gut dysbiosis [19]. At the molecular levels, depletion of colonic SIRT1 has been linked to the activation of several pro-inflammatory pathways including PI3K/AKT and NF-κB [7,8,19,21]. Interestingly, the current data demonstrated the competence of REB to activate the SIRT1/FoxO3a/Nrf2 axis, culminating in improved colonic outcomes. This is consistent with the notion that SIRT1 activation is linked to the alleviation of colonic injury in experimental IBD models—including AA-induced colitis—culminating in improved colitis symptoms [8,19]. Mechanistically, SIRT1 deacetylates/activates the downstream signal FoxO3a resulting in dampened ROS production and lowered mitochondrial damage [46]. FoxOs are also essential for cell survival via transactivating several cellular antioxidants including SOD [13]. Moreover, SIRT1 activates the antioxidant Nrf2/HO-1 pathway resulting in dampened cellular oxidative stress and apoptosis [19,23]. SIRT1 also suppresses colonic inflammation via the inhibition of the NF-κB pathway likely via direct deacetylation of NF-κBp65 at lysine 310 [21]. 

Downregulation of Nrf2 and its downstream antioxidant signals has been linked to the exacerbation of colonic oxidative injury [7,23,34]. Consistent with these data, the present findings revealed that AA-triggered the inhibition of Nrf2 antioxidant signal and depleted the colonic antioxidant defenses by inhibiting GPx, GST, SOD, and CAT enzymes. Interestingly, REB demonstrated favorable outcomes on colonic oxidative stress by dampening TBARS together with the stimulation of Nrf2. In fact, the transcription factor Nrf2 is crucial for controlling the synthesis of several antioxidant enzymes such as GPx and SOD. Generally, activation of Nrf2 provokes crucial protective effects against IBD consequences [23,47]. Notably, the interplay between Nrf2 and NF-κB has been previously characterized. In this regard, the diminished cellular Nrf2 has been associated with enhanced NF-κB transcriptional activity, culminating in overshooting of pro-inflammatory cytokines [48]. Hence, the observed upregulation of Nrf2 protein expression and the downregulation of Keap-1 expression by REB confirms its efficacy in curbing the colonic oxidative insult and enhancement of colon antioxidant signals. 

The crosstalk among SIRT1, Nrf2, and FoxO3a has been characterized to be tightly controlled. Evidence exists that SIRT1 deacetylates Nrf2, resulting in enhanced Nrf2 protein expression [49]. In the same context, SIRT1 upregulates the expression of the cytoprotective FoxO3a and its target genes and dampens the transcription of apoptosis genes, thereby favoring cell survival. Of note, the activity of the transcription factor FoxO3a is also controlled by PI3K/AKT pathway [35]. Evidence exists that PI3K/AKT pathway modulates FoxO3a activity by phosphorylation resulting in its inactivation. Consistently, the current data revealed that REB inhibited PI3K/AKT pathway and activated SIRT1/FoxO3a pathway, culminating in improved colonic outcomes and cell survival. In the same regard, the interplay between SIRT1 and NF-κB has been highlighted. In this regard, SIRT1 has been reported to suppress NF-κB pathway and associated pro-inflammatory cytokine production [7,35]. The latter observation is mediated through SIRT1-induced suppression of NF-κB p65 nuclear translocation. In the same context, the cytoprotective signal PPAR-γ has been reported to suppress NF-κB pathway via deacetylation/inactivation of NF-κBp65 [50]. In fact, upregulation of PPAR-γ has been proven to protect against UC induced by AA. In the colonic microenvironment, an enhanced protein expression of PPAR-γ has been reported to abrogate NF-κB pathway and enhance the antioxidant status in the colonic tissue [51,52]; thereby mitigating the colitis symptoms [18]. These data, when taken collectively, may provide a molecular viewpoint on REB’s protective function against UC via the activation of SIRT1/FoxO3a/Nrf2 axis alongside inhibition of the pro-inflammatory PI3K/AKT pathway.

## 4. Materials and Methods

### 4.1. Chemicals

Rebamipide and acetic acid were purchased from Sigma Aldrich (St. Louis, MO, USA) and El-Naser Pharmaceutical Chemicals Company (Cairo, Egypt), respectively.

### 4.2. Experimental Animals

Male Wistar rats (200–220 g) were supplied from the Faculty of Medicine at Assiut University and housed under standard conditions with a standard feed and unlimited access to drinking water. All experimental procedures were approved by the Animal Ethics Committee at the Faculty of Medicine, Assiut University (IRB No.17300461) and were in line with the guidelines of the National Institutes of Health (NIH) for the Care and Use of Laboratory Animals (publication No. 85–23, revised 1985). 

### 4.3. Induction of Colitis 

Before colitis induction, rats were kept without food for one day but with free access to drinking water. Ketamine was used as an anesthetic (100 mg/kg, i.p.). Then, 2 mL of acetic acid (AA; 3% *v/v* in saline) was administered intrarectally using an elastic catheter to induce colitis. The catheter was inserted into the colon up to 8 cm rectally and 2 mL of air was injected afterward to completely spread the AA throughout the colon. To prevent AA leakage, rats were kept in a head down/straight position for 2 min. Both control and REB-treated control rats were intrarectally injected with saline (2 mL) instead of AA [36]. 

### 4.4. Experimental Design

**Figure 12:**
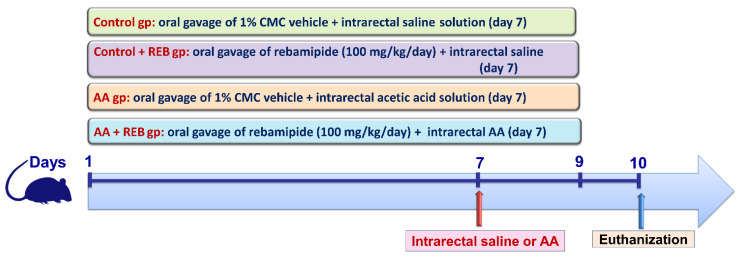
Schematic outline of the experimental study design. REB, rebamipide; AA, 3% acetic acid solution (3% *v*/*v* in saline).

Rebamipide was suspended in 1% carboxymethyl cellulose (CMC) and AA was prepared as a 3% solution in saline (*v*/*v*). As illustrated in Figure 12, 32 rats were randomly allocated into 4 groups of 8 rats each. Group I (Control rats): received 1% CMC for nine days as a vehicle by oral gavage and 2 mL of saline solution was rectally infused on the seventh day instead of AA. Group II (Control + REB group): received rebamipide (REB; 100 mg/kg/day) suspended in 1% CMC [29,53] once daily for nine days by oral gavage and 2 mL of saline solution was rectally infused on the seventh day instead of AA. Group III (AA group): received 1% CMC for nine days as a vehicle by oral gavage and received a rectal infusion of 2 mL AA (3% *v*/*v* in saline) on the seventh day. Group IV (REB + AA group): received REB (100 mg/kg/day) suspended in 1% CMC [29,53] once daily for nine days and received a rectal infusion of 2 mL AA (3% *v*/*v* in saline) on the seventh day. The animals were euthanized on the 10th day. Notably, the selected dose of rebamipide is consistent with previous literature that revealed the efficacy of rebamipide for dampening inflammation in rodent models of indomethacin-induced intestinal damage [53], ethanol-induced gastric mucosal damage [26], methotrexate-evoked hepatic injury [29], and 6-hydroxydopamine-induced Parkinson’s features [30].

### 4.5. Sample Preparation

Ketamine (100 mg/kg, i.p.) was used to anesthetize rats; then, blood was withdrawn through heart puncture 24 h following the last dose. The sera were prepared from blood samples and then centrifugated at 1000× *g*. The colon was dissected, washed with saline, and examined macroscopically for scoring (colon mucosal injury score; CMIS). The Colon was then cut into four small pieces. In one part of the colon, histology was applied, and immunohistochemistry was used to assess the protein expression of PI3K, p-AKT(Ser473), Nrf2, and PPAR-γ. The second part was kept in RNA*later^®^* solution for determining the gene expression of *Keap-1*, *Nrf2*, *NF-κB*, *SIRT1*, *FoxO3a*, *PPAR-γ*, and glyceraldehyde-3-Phosphate dehydrogenase (*GAPDH*) genes using qRT-PCR. The third part was used to prepare homogenates of 20% (*w*/*v*) in cold saline for oxidative stress markers while the fourth part was homogenized in protease inhibitor-complemented RIPA buffer for the ELISA assays.

### 4.6. Disease Activity Index (DAI) and Macroscopic Damage Scoring 

Colitis severity was determined by the disease activity index (DAI) using a 0–12 scoring system [38]. For weight loss, points were distributed from 0 to 4. Moreover, the stool samples were collected, examined, and scored from 0 to 4 (for the diarrhea score). For occult blood, points were distributed from 0 to 4. The total score of DAI was calculated by the following formula: DAI = Body weight loss score + diarrhea score + rectal bleeding score. In addition, the macroscopical damage was evaluated using a 0–5 scoring system and was expressed as the colon mucosal injury score (CMIS) as previously described [54]. The criteria for the colon mucosal injury score are listed in Table 1. For both parameters, the identity of experimental groups was kept anonymous to avoid potential bias.

### 4.7. Histopathology and Microscopical Damage Scores

Three colon specimens were taken randomly from three animals per experimental group. The colon tissue was fixed overnight in a 10% neutral buffered formaldehyde solution before being embedded in paraffin, followed by staining with hematoxylin and eosin (H–E) for examination [34,55]. In each group, six non-overlapping fields were imaged. The histological images showing colon morphology were obtained by microscopy (Leica Microsystems, Wetzlar, Germany). The specimen identity was kept anonymous during image capturing and analysis to avoid potential bias.

The colon microscopical damage scores were used to quantify the histopathological aberrations in the colons according to a 0–5 scoring protocol [56]. Herein, a score of 0 described a lack of specific changes; a score of 1 described focal inflammation/ulceration located in the mucosal layer; a score of 2 described focal or extensive inflammation/ulceration located in the mucosa and submucosa layers; a score of 3 described focal or extensive inflammation/ulceration located in the mucosa, submucosa, and muscularis layers; a score of 4 described focal inflammation/ulceration spanning all colon layers till the serosa layer; a score of 5 described extensive inflammation/ulceration spanning all layers including the serosa layer.

### 4.8. Measurement of TNF-α, IL-6, and CRP

Serum levels of tumor necrosis factor-alpha (TNF-α) and interleukin-6 (IL-6) were assessed biochemically using rat-specific ELISA kits (Cat. No. E-EL-R2856 and E-EL-R0015, respectively, Elabscience, Wuhan, China). Moreover, the C-reactive protein (CRP) was assessed using an ELISA kit from Elabscience (Cat. No. E-EL-R0506, Elabscience, Wuhan, China) following the guidelines of the manufacturer.

### 4.9. Assessment of Colon Oxidative Stress Markers

The colonic content of lipid peroxides expressed as thiobarbituric acid reactive substances (TBARS) was assayed using the assay established by Mihara and Uchiyama [57]. Briefly, the assay was based on the reaction between lipid peroxides and thiobarbituric acid (TBA) to produce a pink color. The tissue homogenate was mixed with 1% O-phosphoric acid, and 0.6% TBA, and the mixture was heated for 45 min at 95 °C in a water bath. After cooling, n-butanol was added, the butanol phase was separated by centrifugation at 1000× *g*, and the optical density of the pink color was measured at 535 and 520 nm. The assay of colonic reduced glutathione (GSH) was applied using the Ellman method [57]. Herein, the assay was based on the reaction between the sulfhydryl group of the reduced glutathione and 5, 5′-dithio-bis (2-nitrobenzoic acid) to yield a yellow-colored complex (nitro-mercaptobenzoic acid). The optical density of the yellow color was measured at 412 nm. 

The assay of glutathione peroxidase (GPx) enzyme was applied according to the method established by Lawrence, et al. [58]. Herein, GPx generates an oxidized form of glutathione which reacts with NADPH.H^+^ in a reaction catalyzed by glutathione reductase resulting in the formation of NADP^+^. As a result of decreased levels of NADPH.H^+^, the resultant decline in the optical density was monitored kinetically at 340 nm. Moreover, the assay of glutathione-S-transferase (GST) enzyme was applied according to the method established by Keen, et al. [59]. To this end, the homogenate supernatant was mixed with 0.1 M phosphate buffer (pH 6.5), GSH, and 2,4-dinitrochlorobenzene. Kinetic monitoring of the optical density was performed at 340 nm. Finally, catalase (CAT) enzymatic activity assay was applied according to the method established by Sinha [60]. Herein, CAT activity was determined based on the decline in the absorbance at 240 nm in response to the decomposition of H_2_O_2_ by CAT enzyme. 

### 4.10. Immunohistochemical Analysis 

Three colon specimens were taken randomly from three animals per experimental group. Colon sections with a thickness of 4 μm were dehydrated in xylene first then in ascending solutions of ethanol [61,62]. The antigen retrieval step was applied using a citrate buffer (pH 6.0) in a microwave, and 5% bovine serum albumin in Tris-buffered saline was used to block the sections for 2 h. Then Nrf2, PPAR-γ, PI3K, and p-AKT primary antibodies with Cat. No. YPA1865, YPA2204 (1:50 dilution; Chongqing Biospes Co., Chongqing, China China), E-AB-64202 (1:100 dilution; Elabscience, Wuhan, China), and sc-514032 (1:50 dilution; Santa Cruz Biotechnology, Dallas, TX, USA), respectively, were incubated overnight. Negative control sections were processed similarly except for the omission of the primary antibody incubation step. Hematoxylin counterstaining was applied, and the slides were examined. Tissues were examined for immune positivity staining by light microscopy (Leica Microsystems, Wetzlar, Germany). In each group, six non-overlapping fields were imaged. The percentage of the positive areas with brown staining in the digital images was calculated using the Fiji ImageJ^®^ (version 1.51r; NIH, Bethesda, MD, USA) software, and each image’s average area was calculated. To this end, the average area of the immunostaining was measured by applying the color deconvolution 2 plugin to allow the separation of the initial RGB image into three 8-bit images. Color analysis was performed on the DAB image. This allows the measurement of the brown-colored areas specific to immunostaining while discarding other dark areas in images. The specimen identity was kept anonymous during image capturing and analysis to avoid potential bias. 

### 4.11. Quantitative Real-Time PCR Analysis

The gene expression levels of *Keap-1*, *Nrf2*, *NF-κBp65*, *SIRT1*, *FoxO3a*, *PPAR-γ*, and *GAPDH* mRNA expression were measured using qRT-PCR. The primers for these genes were purchased from Vivantis Technologies Sdn. Bhd, Selangor Darul Ehsan, Malaysia. Colonic RNA was extracted using the Trizol reagent (Invitrogen Inc., Grand Island, NY, USA) and the RNA purity was checked using absorbance at 260 nm and 280 nm (samples with A260/A280 ranged from 1.8 to 2). Then, 1 µg of RNA was reverse transcribed to cDNA (Thermo Scientific Revert Aid, Waltham, MA, USA) as recommended by the manufacturer. As previously described, SYBR green (Bioline, myBio, Dublin, Ireland) was employed to test target gene expression [63]. Using the GAPDH as the housekeeping gene, the 2^−ΔΔCT^ equation was applied and the data obtained after PCR amplification were expressed as a fold change, as described by Livak and Schmittgen [64]. Sequences of primers are listed in Table 2.

### 4.12. Statistical Analysis

GraphPad Prism Software (Version 7.00) was used to run statistical analyses. The normality and homogeneity of the values were examined by the Shapiro–Wilk test. For normally distributed (parametric) values, a one-way ANOVA followed by a Tukey’s test post hoc analysis was applied to detect statistical significance among experimental groups. The parametric data were presented in the form of mean ± standard deviation (S.D.). For non-parametric values (DAI, CMIS, and histology scores), the Kruskal–Wallis test followed by the Dunn’s multiple comparison post-test were applied to detect statistical significance. The non-parametric data were presented in the form of median with the interquartile range. The statistical significance was defined at *p*-value of less than 0.05.

## 5. Conclusions

In conclusion, the present study described some molecular mechanisms for the ameliorative effects of REB against experimental UC. In the colon microenvironment, these beneficial effects were mediated by suppressing the inflammatory response and oxidative stress via curbing PI3K/AKT pathway alongside the stimulation of SIRT1/FoxO3a/Nrf2 axis and PPAR-γ cytoprotective signal. Therefore, the present data may add to the current knowledge regarding the underlying molecular mechanisms of REB against the pathogenesis of UC. Additionally, they may provide a rationale for using REB as an adjunct modality for ulcerative colitis particularly in patients with concomitant gastric ulcers. Yet, clinical data is warranted to prove that point. Notably, supplemental studies are also required to characterize the detailed molecular pathways involved in REB’s actions including examination of the ratio of phosphorylated/total AKT. 

## Figures and Tables

**Table 1:** Colon mucosal injury scoring criteria.

Score	Criterion
0	No injury
1	Mild hyperemia with a tiny portion of edema and no erosion or ulceration
2	Moderate hyperemia with moderate edema and one erosion site
3	Moderate hyperemia with moderate edema and two erosion sites
4	Severe hyperemia with severe edema, and inflammation covering the mucosal layer, and with major inflammation not exceeding 1 cm in diameter
5	Severe hyperemia with severe edema, swelling, bleeding, and inflammation covering the mucosal layer, and with major inflammation measuring more than 1 cm in diameter.

**Table 2:** Primer list for qRT-PCR.

Target Gene	Gene Accession Number	The Nucleotide Sequence (5′-3′)
*NF-κB*	XM_006233360.4	F: TGGGACGACACCTCTACACA R: GGAGCTCATCTCATAGTTGTCC
*Nrf2*	NM_001399173.1	F: ATTGCTGTCCATCTCTGTCAG R: GCTATTTTCCATTCCCGAGTTAC
*Keap-1*	XM_006242591.3	F: TCAGCTAGAGGCGTACTGGA R: TTCGGTTACCATCCTGCGAG
*PPAR-γ*	NM_001145367.1	F: GGGACGCTGAAGAAGAGACCTG R: CACAGTCCGGTCAGAAAGTGA
*SIRT1*	NM_001414959.1	F: CGGTCTGTCAGCATCATCTTCC R: CGCCTTATCCTCTAGTTCCTGTG
*FOXO-3*	NM_001106395.1	F: GCCTCATCTCAAAGCTGGGT R: AGTTCTGCTCCACGGGAAAG
*GAPDH*	NM_017008.4	F: TGCTGGTGCTGAGTATGTCG R: TTGAGAGCAATGCCAGCC

## Data Availability

Data are contained within the article.
